# Exploring Vector-Borne Disease Surveillance and Response Systems in Beijing, China: A Qualitative Study from the Health System Perspective

**DOI:** 10.3390/ijerph17228512

**Published:** 2020-11-17

**Authors:** Jerome Lock-Wah-Hoon, Yang Zheng, Marieta Braks, Liselotte van Asten, Qiyong Liu, Preeti Sushama, Simone Doreleijers, Thomas Krafft, Wim van der Hoek, Ewout Fanoy, Quanyi Wang, Eva Pilot

**Affiliations:** 1 Department of Health, Ethics and Society, Care and Public Health Research Institute (CAPHRI), Faculty of Health, Medicine and Life Sciences, Maastricht University, 6229HA Maastricht, The Netherlands; j.lock-wah-hoon@maastrichtuniversity.nl (J.L.-W.-H.); preeti.sushama@maastrichtuniversity.nl (P.S.); s.doreleijers@maastrichtuniversity.nl (S.D.); thomas.krafft@maastrichtuniversity.nl (T.K.); 2 Institute for Infectious Disease and Endemic Disease Control, Beijing Centre for Disease Control and Prevention, Beijing 100013, China; yoyozheng1982@163.com (Y.Z.); bjcdcxm@126.com (Q.W.); 3 Centre for Infectious Disease Control Netherlands, National Institute for Public health and the Environment (RIVM), 3720 BA Bilthoven, The Netherlands; marieta.braks@rivm.nl (M.B.); liselotte.van.asten@rivm.nl (L.v.A.); wim.van.der.hoek@rivm.nl (W.v.d.H.); 4 Department of Vector Biology and Control, National Institute for Communicable Disease Control and Prevention, Chinese Center for Disease Control and Prevention, Beijing 102206, China; liuqiyong@icdc.cn; 5 Department of Infectious Diseases, Public Health Service Rotterdam-Rijnmond, 95 3011 EN Rotterdam, The Netherlands; eb.fanoy@rotterdam.nl

**Keywords:** vector-borne disease, public health, health information systems, international health regulations, outbreak, Beijing, China

## Abstract

Background: Climate change may contribute to higher incidence and wider geographic spread of vector borne diseases (VBDs). Effective monitoring and surveillance of VBDs is of paramount importance for the prevention of and timely response to outbreaks. Although international regulations exist to support this, barriers and operational challenges within countries hamper efficient monitoring. As a first step to optimise VBD surveillance and monitoring, it is important to gain a deeper understanding of system characteristics and experiences in to date non-endemic regions at risk of becoming endemic in the future. Therefore, this study qualitatively analyses the nature and flexibility of VBD surveillance and response in Beijing. Methods: In this qualitative study, eleven experts working in Beijing’s vector-borne diseases surveillance and response system were interviewed about vector-borne disease surveillance, early warning, response, and strengths and weaknesses of the current approach. Results: Vector-borne disease surveillance occurs using passive syndromic surveillance and separate vector surveillance. Public health authorities use internet reporting networks to determine vector-borne disease risk across Beijing. Response toward a vector-borne disease outbreak is uncommon in this setting due to the currently low occurrence of outbreaks. Conclusions: A robust network of centralised institutions provides the continuity and flexibility needed to adapt and manage possible vector-borne disease threats. Opportunities exist for population-based health promotion and the integration of environment and climate monitoring in vector-borne disease surveillance.

## 1. Introduction

### 1.1. Governing Global Health: Surveillance for Vector-Borne Diseases

Vector-borne diseases (VBDs) account for more than 17% of global infectious disease burdens and contribute to emerging and re-emerging disease threats, causing concern within the global public health community [1]. VBDs present with complex transmission dynamics, influenced by multiple interconnected indicators including the climate, environmental variation, and vector and pathogen distribution [2]. The Intergovernmental Panel on Climate Change highlights climate change as an important risk factor to global health security, including the associated distribution and occurrence of VBDs [3]. Research suggests that climate change will lead to a higher incidence and wider geographic spread of VBDs in explicit regions by the disruption of complex ecological systems that determine vector and pathogen distribution, contributing to uncertain future scenarios [4,5]. Establishing effective VBD monitoring and surveillance is of paramount importance for the prevention of and timely response to VBD outbreaks now and in the future.

Through the International Health Regulations (IHR) (2005), endorsing countries have agreed to build their capacities to detect, assess, and report public health events to strengthen global health security [6]. State parties are required to carry out assessment of public health events within their borders that can develop into a public health emergency of international concern. Annex 2 acts as a decision-making framework for the assessment and notification of such events, delivering core elements of a surveillance and response system (SRS) [6]. It seeks integration of core attributes—usefulness, sensitivity, timeliness, and stability into health systems [7]. The IHR contain specific measures toward VBDs, emphasising syndromic surveillance to expedite reporting [8]. Research conducted on the value and effectiveness of the IHR conclude that reported operational difficulties are likely due to barriers within countries. These include poor surveillance infrastructure, poor data and/or information flow, and political barriers due to perceived restrictions to travel and trade [9].

### 1.2. China after the 2003 SARS Epidemic

In the past decades, China has emerged as a global economic power and an important actor in global health [10]. In agreement to the IHR and to lessons learned from the 2003 SARS epidemic and recently Covid-19, China advanced core surveillance capacities to detect, assess, and report health events that threaten national and global health security in a timely manner [11]. Consequently, Chinese public health experienced important changes where policy development in support of public health resulted in stern and strenuous efforts towards progressive healthcare reforms leading to increased governmental commitment to public health. As more resources flow into public health activities, the prevention and control of disease is worked towards with surveillance systems greatly improved. The use of modern information technologies in reporting networks is a key development that strengthens public health systems [12,13]. The China Infectious Disease Automated-alert and Response System (CIDARS), coordinated by the national China Center for Disease Prevention and Control (CDC), represents a web-based reporting network that has been integrated into routine surveillance efforts at all public health governance levels [14,15]. Notification compliance of public health bodies exceeds 90% [16]. Utilising passive and active reporting, including laboratory and syndromic surveillance, internet reporting networks have transformed how data are collected and analysed, supporting timely response to and prevention of VBD outbreaks [17]. Computer-based modelling using geographic information systems is also applied to form thematic digital maps. These maps reveal spatial, temporal, and predictive distributions of disease cases and vector populations to inform SRS activities [18,19].

### 1.3. Beijing and Vector-Borne Diseases

Beijing is currently non-endemic for many VBDs. However, Beijing municipal province experiences rapid urban growth and represents an international hub for travel and trade. It accommodates a growing population of about 22 million, causing large demands for basic services including transport, education, water, energy, and healthcare [20]. Patterns of resource consumption drive rapid environmental and land-use change, affecting the regional climate, ecology of vectors and hence the regional distribution of VBDs [21]. Fourteen tick and four mosquito species are present in the municipality of Beijing. Their distribution is influenced by dynamic global, social, environmental, and meteorological factors of Beijing’s urban and rural districts [22,23]. These features form risk factors for Beijing to become endemic for VBDs in the future, with potentially large impacts on human health in the municipality.

### 1.4. Aim of the Study

Although previous studies have described Chinese infectious disease surveillance [15], further detailed/in depth study of the local efficacy and adaptability of VBD surveillance in Beijing province is of importance to enhance preparedness for prospective developments in VBD occurrence. Despite the existence of international regulations to support adequate monitoring systems, barriers and operational challenges within countries can hamper efficient monitoring. Therefore, this study aims to qualitatively explore experiences, notions and perceptions of local public health experts concerning the current practice of VBD surveillance. The study specifically focuses on system flexibility to explore how Beijing’s VBD surveillance orientates itself to manage possible arboviral outbreaks. A deeper understanding of perceived strengths and weaknesses of the system may identify existing gaps in surveillance and serve as a fundament for system enhancement. Further, the system analysis can help other regions to tailor their surveillance and response to VBD.

## 2. Methods

**Figure 1:**
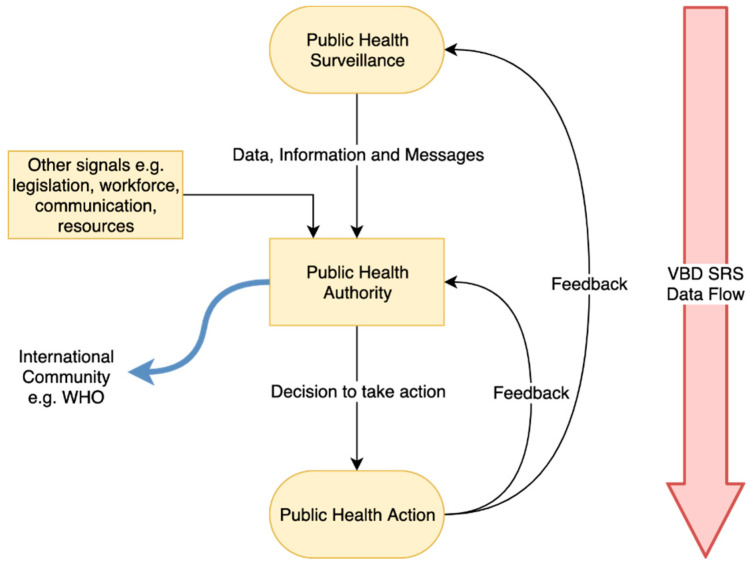
Vector Borne Disease Surveillance and Response System framework. Source: Adapted from McNabb et al. (2002) [24].

An exploratory qualitative study design was adopted to understand VBD SRS as understood by government experts from the main public health institution responsible for public health within the province. The data collection took place between June and August 2018, and 11 public health experts specialised in VBD and SRS were interviewed. All respondents were employed in the municipal province of Beijing. A vector borne disease surveillance and response system framework was adapted from the conceptual framework for public health surveillance and action of McNabb et al. (2002) [24], which focuses on dataflow between public health surveillance and public health authorities, and on central processes between both ends that influence the decision-making capacity of public health authorities. Examples of interfering factors include national and international legislation, workforce, communication and available resources. Data on the evaluation of control measures are subsequently fed back into the surveillance system to support any required changes. This model aims to frame the constructivist reality of Beijing’s VBD SRS this paper (Figure 1).

### 2.1. Sampling

Eleven respondents (hereafter referred to as experts), R1 through to R11, were identified through purposive sampling facilitated by key informants and convenience sampling. We included professionals with different backgrounds from epidemiology, clinicians with expertise on VBD, entomologist, public health surveillance system experts. Experts were invited via email and phone call to participate in the study. Inclusion to the study concentrated on their expertise, area of work, and direct involvement in VBDs and SRS. In light of system representation, experts at the district, provincial, and national levels, including urban and rural areas, were involved. In Table 1 the characteristics of participants are further elaborated on.

### 2.2. Data Collection

Semi-structured one-to-one interviews were conducted in English, with an interpreter available in instances where experts preferred to converse in Chinese Mandarin. The data collection (interview guide) on VBD surveillance and response system characteristics in Beijing province were guided based on integrated approach towards surveillance of vector-borne diseases by Braks et al. (2011) [2] and the CDC’s Updated Guidelines for Evaluating Public Health Surveillance Systems [25]. See Supplementary Material 1.

Interviews were recorded and generally lasted for one hour.

### 2.3. Data Analysis

Audio-recorded interviews were transcribed with all personal information excluded. Thematic content analysis was performed using ATLAS.ti qualitative analysis software (ATLAS.ti Scientific Software Development GmbH, Berlin, Germany). Themes aligned to the adapted framework and questionnaire. Prior to coding and analysis, transcripts were reviewed to establish a general tone. A set of themes of discourse present in the expert’s narrative accounts was developed and aligned to key framework themes. These were consolidated to form initial codes. Primary codes represented the larger context of VBD SRS, e.g., monitored indicators, guidelines, research actions. Secondary codes were used to contextualise the SRS reality, e.g., to suggest underlying experiences including system strengths and opportunities for development. An iterative process followed to consolidate findings.

### 2.4. Ethics and Consent

The entire research involves official and published data, as well as information given by experts with informed consent. The ethics review board at Maastricht University approved the study in May 2018, with no further ethical reservations. Further, the standards of ethical conduct outlined by the Netherlands Code of Conduct for Research Integrity and further agreed on under the collaboration agreement between the main collaborating institutions (UM, CDC, RIVM) were followed.

## 3. Results

### 3.1. General Organisation and Administration of VBD Surveillance in Beijing

Disease and vector surveillance operates to a top-down governance framework, though lower level authorities have the autonomy to manage their respective work on the condition of keeping within the scope of national level instruction and guidelines. The collected surveillance data flows in a vertical bottom to top reporting structure and is stored in a central database.

*“We use Internet portals for surveillance in Beijing, especially the District CDC’s. They collect surveillance data and report this to Beijing CDC. Beijing CDC will collect the district level data and report this directly to the national China CDC.” [R8]*

**Figure 2:**
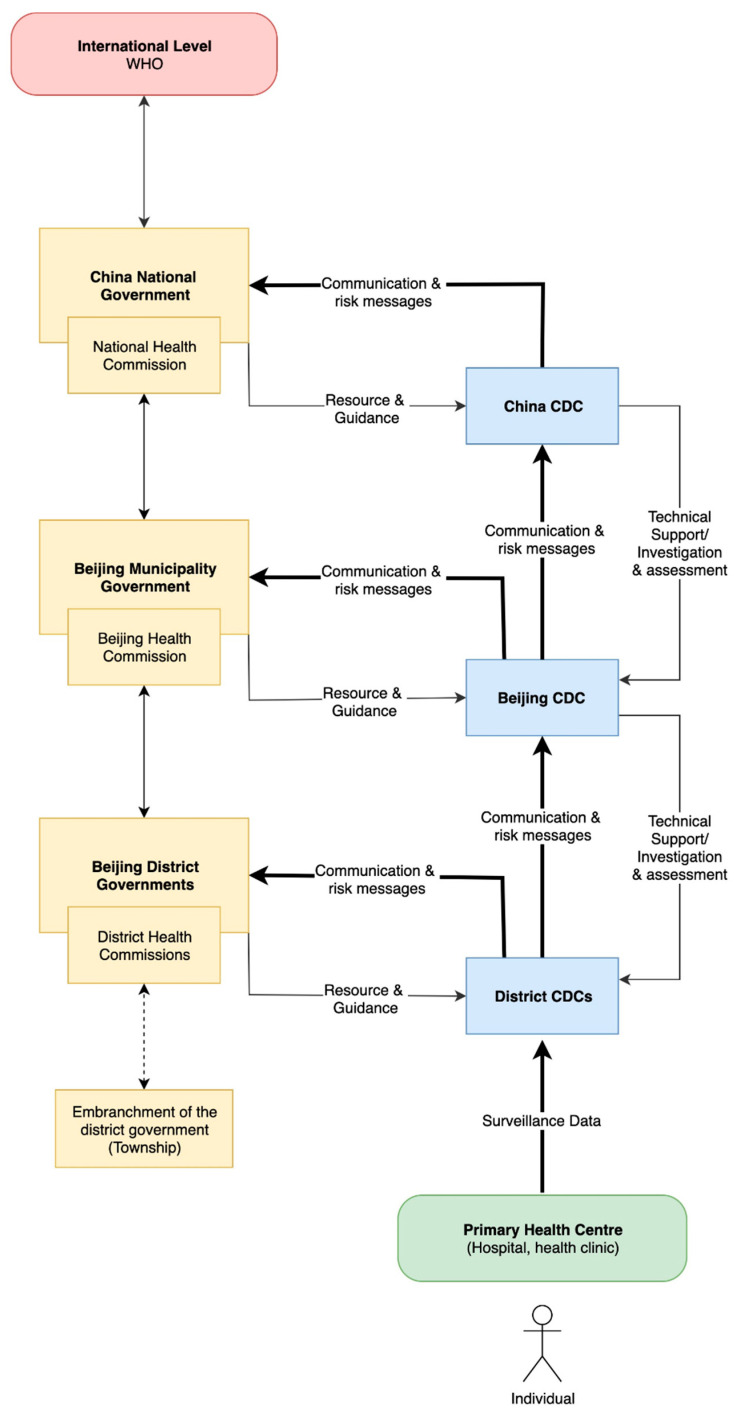
Simplified system map of authorities involved in vector-borne disease surveillance and response in Beijing Municipality. Green box represents the primary care centre and the entry point of the individual into the healthcare system. Yellow boxes represent administrative health authorities. Blue boxes represent technical health authorities. Interactions between authorities are indicated using black arrows. This system map was developed based on qualitative data from experts from Beijing.

Two kinds of public health authorities are responsible for epidemiological surveillance data. Administrative public health authorities, specifically the Municipal Health Commission, guide resource distribution and system activities. Technical public health authorities comprising district, provincial, and national level CDCs provide expert technical support and direction for population health management. Both health authorities analyse collected surveillance data to conduct risk assessments, revise monitoring guidelines, and form a basis for public health messages. Their analysis supports control actions for both human VBD cases and vectors that transmit pathogens (Figure 2). Technical authorities perform surveillance activities focusing on disease and vector monitoring. Environmental and climate monitoring are not integrated into routine surveillance activities, but official meteorological data are available for use by technical authorities.

**Figure 3:**
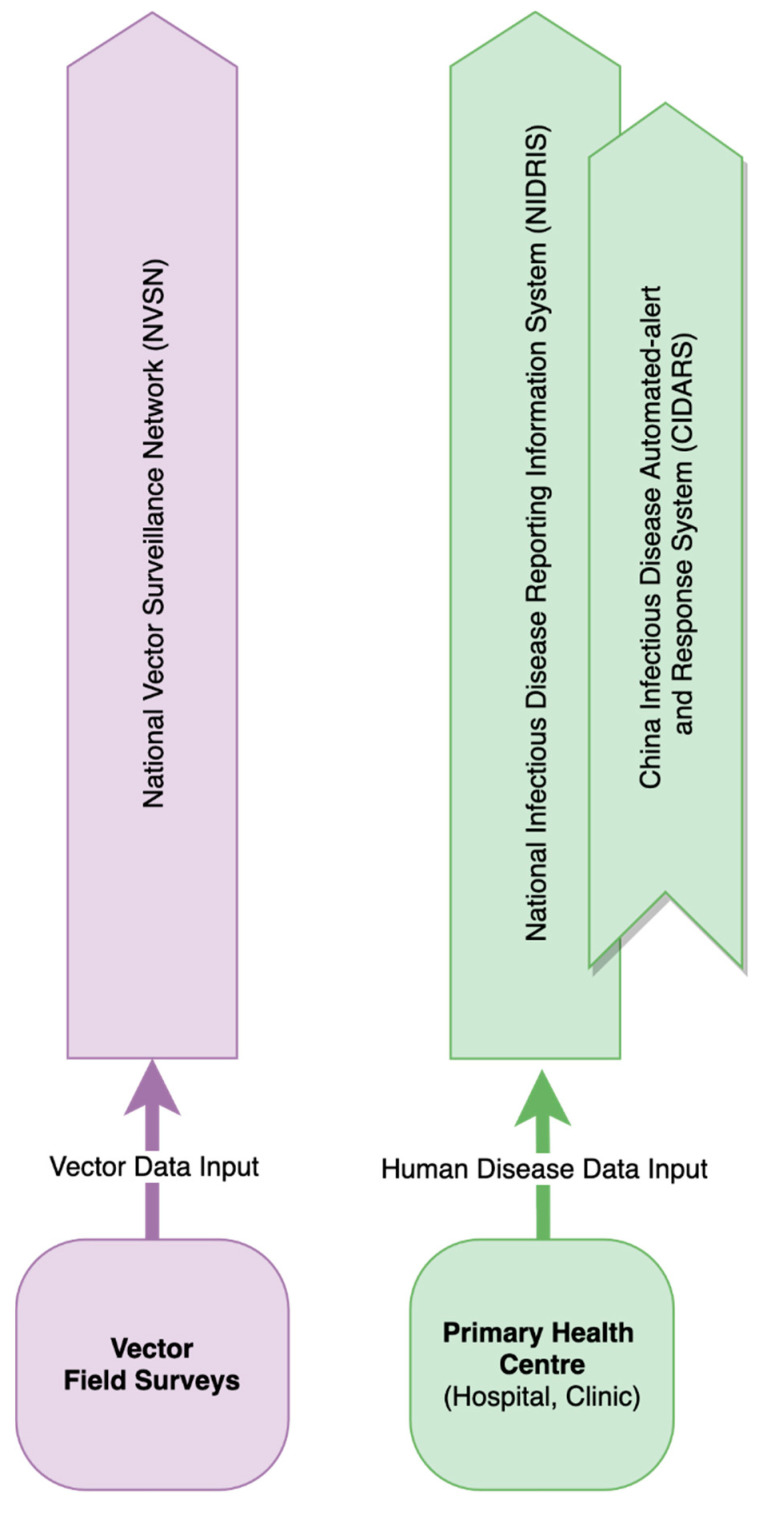
Representation of web-based reporting networks developed by China CDC. Purple represents vector web-reporting structures. Green represents human disease case web-reporting structures. CIDARS using data from NIDRIS acts as a real-time alert system using SMS to notify authorities of abnormal data signals. Health authorities at all governance levels are able to review the data to support response actions. This representation was based on qualitative data from experts from Beijing.

Human case reporting and vector surveillance operate within separate reporting networks developed at the national level by China CDC, respectively the National Infectious Disease Reporting Information System in China (NIDRIS) and the National Vector Surveillance Networks (NVSN) (Figure 3). NIDRIS and NVSN serve as tools to report information from the field to the public health service. CIDARS uses data from NIDRIS to notify authorities of signals that require evaluation for possible outbreak in real-time through SMS. VBD surveillance data are collected into NIDRIS at district governance levels with provincial and national CDCs able to access the data for evaluation and translation into public health messages. Surveillance activities are managed and directed in the first instance by district level health authorities.

At the primary healthcare level, passive syndromic surveillance is a first layer approach to detect human cases. Clinicians report syndromic cases if they meet the notifiable case criteria, defined as two or more of the following: fever with diarrhoea, respiratory syndrome, bleeding disorder, central nervous system disorder, and rash. Reported data include the patient’s name, date of birth, gender, residential address, date of onset, date of diagnosis and diagnosis result. The surveillance system is largely dependent on a patient’s self-agency to enter the health system at hospital level.

Laboratory testing at reference CDC and hospital laboratories occurs following initial syndromic evaluation, epidemiological inquiry and case registration into NIDRIS. Experts valued the current passive syndromic surveillance system as it enables adequate and quick triage of patients, supporting well-timed reporting and prompt distribution of surveillance data. Reference CDC laboratories are equipped with molecular diagnostic technologies (polymerase chain reaction, viral load) to screen and confirm an infection. Hospitals are improving on-site molecular diagnostic capacities, though shortage of experts specialised in processing genetic sequencing data were reported. Clinical surveillance data from the primary health facility level is transmitted using NIDRIS and flows from the health facility to district authorities. Data are accessed and actioned by provincial, or national level CDCs depending on case severity, disease notification legislation, and perceived public health risk. The Law of Prevention and Control of Infectious Disease in China guides VBD prioritisation; however, VBDs do not pose a significant risk in Beijing where respiratory infections take precedence. The Chinese notifiable disease list contains 42 diseases listed in the Law Of The People’s Republic Of China On Prevention And Treatment Of Infectious Diseases including VBDs that are spread by mosquito and tick species (dengue fever, malaria, Japanese encephalitis, kalaazar) [26].

*“The priority disease in Beijing is influenza and respiratory infections... VBDs are not at the first attention.” [R11]*

Vector surveillance in Beijing is organised across technical and administrative authorities through the NVSN. This is structurally similar to NIDRIS, where district level institutions perform core operational tasks, supported by provincial and national institutions when required. Vector surveillance is catchall and managed by staff of the Vector and Disinfection Department in Beijing’s 16 district CDCs. Beijing’s provincial CDC is focused on research, providing active support when required. Surveillance priority is given to mosquito populations, which increases during the warmer and wetter summer months. Tools and parameters used in mosquito surveillance are the light trap for adult mosquitoes and the load index for larvae present in water bodies.

*“Light trap, to catch mosquito, every month. From July to October we increase frequency to every 10 days.” [R3]*

Public engagement in surveillance activities is not popular in China, but experts noted that citizen involvement in surveillance is useful in other countries.

*“In Mexico, South America, Brazil, maybe in Australia, the community are involved in the vector surveillance, but in China this is not popular. Just the CDC staff carry out vector surveys.” [R9]*

Monitoring mosquito populations in Beijing provides information about density, species, and annual distribution patterns with research activities accompanying information gained from monitoring activities. Data collected from the field are reported in NVSN by CDC staff. Data are then accessed by technical and administrative authorities with the aim of knowing the current status of vector distribution. Reports supplement the dissemination of information gained at district levels. Guidelines and legislation supporting vector surveillance are formed at Beijing’s provincial level and largely depend on data collected.

Municipal and national authorities also monitor abnormal signals to support district level authorities when needed. Airport Inspection and Quarantine Bureaus are involved in mosquito and incoming traveller surveillance at Beijing Capital International Airport due to import risks. For travellers, point of care diagnostic tests such as Malaria rapid tests, temperature screening, and questionnaires about health status and travel history are used. Airport surveillance operates separately to work conducted by centralised CDCs. Organisations outside of Beijing’s centralised public health system are not involved in routine surveillance during non-outbreak situations.

*“In the airport, we have a temperature surveillance system. We also use a lot of point of care tests. The turnaround time for each examination is 2 h, so travelers can be isolated somewhere, only waiting for 2 h. If the test result is positive, the patient is transferred to the hospital.” [R3]*

### 3.2. Characteristics of Vectors and Vector-Borne Disease Surveillance in Beijing

Surveillance system characteristics were explored and are structured according to themes shown in italics.

*Simplicity:* Beijing’s syndromic and vector surveillance system was operationally straightforward with very little difficulty experienced in conducting routine work. Experts attributed this to limited numbers of VBD cases.

*Sensitivity:* Syndrome identification within clinics was deemed sensitive. Experts stated that passive surveillance performed well at the individual level but not at the population level, where hospital identification of syndromes depended on the patient’s decision to seek medical assistance.

*“It is sensitive to find the patients in the hospital, but for the prevalence, it’s not clear. The percentage of the people who have the disease is not clear. So, it is very sensitive to find the disease in a patient, but the ones who are infected with the pathogen in the community, it is not clear because they first need to go to the hospital.” [R6]*

This contributed to perceived gaps in population level data, as experts believed some patients do not readily seek conventional hospital-based healthcare.

*Specificity:* Data were deemed complete and valid. Experts mentioned that taxonomic identification of vectors and clinical case diagnostics is well functioning. This is due to sufficient expertise and diagnostic procedures relying on molecular techniques in CDC reference laboratories.

*Representativeness:* Data present in the system were thought to be relevant and sufficiently utilised.

*Reliability:* Both consistency and reliability of collected data were considered of high quality, although primary care respondents expressed that reliability depended on the value practitioner’s hold toward data reporting. Public hospitals are required to report cases, but this requirement is not comprehensively shared with private hospitals. Experts report that data entry at the hospital level is time consuming for clinicians as administration staff are lacking.

*“Some are reluctant to do such work as it takes a lot of time. After daily clinical work, they have to turn to another computer to input data. It is tedious. It is boring.” [R3]*

*Timeliness:* System timeliness was considered adequate for effective action, although experts indicated that VBDs were uncommon, providing limited opportunity to report. Web reporting systems were perceived to be very beneficial in supporting early warning and response.

*Acceptability:* Government instructions are followed carefully. Clinics expressed positive perceptions to the syndromic surveillance system, with experts aware of their role in patient care.

*Flexibility:* The VBD surveillance system was indicated as flexible and able to adapt to changing conditions based on regionally important VBDs. Mechanisms for change involved submitting requests to the national level, with periodic meetings supplementing decisions to revise the VBD system, but not the national notifiable infectious disease list.

### 3.3. Organisation of VBD Control Actions

Decisions surrounding control actions were made depending on alert level. Beijing’s Municipal Health Commission, supported by CDCs, is the principle decision maker for VBD and vector control. Decisions are informed from situational awareness gathered from consolidated surveillance and epidemiological data. Communication between administrative (municipal health commission) and technical (CDCs) public health authorities supports this. Beijing’s VBD control actions can be grouped into acute outbreak type action and planned management type actions. In case of acute outbreaks, elimination of mosquitoes to interrupt pathogen transmission to humans is prioritised. This is performed within a buffer zone from an identified case. Based on disease and vector information gained from technical authorities, government authorities conduct vector control. The dominant intervention to control mosquito populations in Beijing involves the usage of chemicals or biocides.

*“We eliminate the mosquito to control the patient transmission. Maybe the most important thing is to eliminate the mosquito. We use the insecticide.” [R4]*

Further, environmental management takes place such as dumping of stagnant water from flowerpots and tyres. Human disease control relies on treatment and clinical case management. The inclusion of external private organisations such as pest control companies during outbreaks depend on the capacity and needs of Beijing’s centralised public health institutions. Their involvement is mandated should resources be stretched.

*“In special times, if we can’t send out so many people to carry out the surveillance, we have to get help from the pest control company and other staff.” [R4]*

Regarding planned management actions towards outbreaks, experts saw public health education and promotion for citizens and training for the health workforce as an important pre-emptive system action. Specifically, trainings about exposure reduction to mosquito-borne diseases were thought to be useful. Examples included lifestyle actions around household water management and bite protection.

*“We have public education to eliminate water hazards like emptying flowerpots and tyres of standing water.” [R4]*

Education for the health workforce could be centred on the dissemination of bulletins and awareness materials from health authorities. Beijing’s health system also conducts vaccine campaigns and education programs domestically and abroad for yellow fever, Japanese encephalitis and malaria.

### 3.4. Characteristics of VBD Control Actions

System organisation, communication, and division of tasks fall under government control. Collaborations occur between administrative and technical authorities and can be supplemented by pest control organisations, civil workplaces, and local citizens in a ‘multi-front’ manner. This is in contrast to non-outbreak conditions, where limited to no inter-organisational and civil collaborations occur. Communication of possible VBD outbreak is shared with the public through newspapers, radio broadcasts, and television. A dedicated news spokesman in Beijing’s Family Planning and Health Commission or the CDC itself shares announcements to the press. During outbreak conditions, disease specific guidelines are available to public health workers, formed at the international, national, and provincial governance levels. For example, response guidelines for dengue and Japanese encephalitis are found at the national level, and may be used in Beijing should an outbreak occur. However, when focusing on non-endemic (not notifiable) VBDs in Beijing such as West Nile fever and Lyme disease, no predefined outbreak plan exists but guidelines for rapid outbreak control would be developed promptly in times of an outbreak. Nevertheless, for Lyme disease, a gap was found where the system identifies very few patients but research identified high prevalence of antibody in rural populations.

*“For Lyme we just do scientific research. We do not do further work as it is not a notifiable disease and we have no the fee, to support our surveillance.” [R5]*

Early warning was felt to occur for non-endemic VBDs such as Crimean Congo haemorrhagic fever and West Nile fever relying on syndromic surveillance, though targeted outbreak response plans are not predefined. These VBDs would receive “stronger attention” should an outbreak occur.

*“West Nile is not reportable in China. Maybe it is imported risk. If there is West Nile in Beijing, first we change the guideline to create a new plan for west Nile to complete this surveillance.” [R10]*

Planned management procedures were dominant for vector control, with authorities having pre-formulated guidelines present. Respondents felt that health system finance and workforce resources were adequate for potential outbreak response and noted that extra funding is available from local and national government when required. Experts reported that response actions toward VBD in Beijing were effective and timely, as exemplified by the response to Zika cases in 2016.

*“In Beijing we received maybe 13 or 15 imported Zika cases. The people and government were tense. Our Beijing CDC made good surveillance and control, as well as good guidelines. There was no local transmission so control measures are effective in Beijing.” [R9]*

### 3.5. Communication and Feedback Mechanisms of Beijing’s VBD SRS

Executive summaries and briefings in the form of official reports are directed to district administrative authorities, whilst raw data sets are directed to and accessed by district technical authorities. The reports are available at higher governance levels when required to identify, support and facilitate the control of possible VBD outbreaks when conditions extend beyond the scope of district CDCs. The feedback of information to lower levels of the surveillance system occurs through periodic consultations and phone calls.

*“By phone, by the network… but whenever there is an emergency happening, we directly inform. We give a call, as this is most direct.” [R3]*

Hereby, feedback implementation supports early warning, timely response and provincial and national level policy adaptations. Periodic consultations are used as a communication process where changes in the nature of VBDs are communicated as well as changes to support the well running of the surveillance system itself. This occurs between administrative and technical authorities across all governance levels, as well as hospitals.

*“To change our guidelines or work processes if we find that conditions change or VBDs increase, we make suggestions to change surveillance though expert consultation in a meeting.” [R4].*

### 3.6. Beijing in an International Sphere

Considering the role imported VBDs hold to public health, the Family Planning and Health Commission conducts vaccine campaigns for yellow fever and broad VBD testing abroad. Here, vaccines are delivered and administered to Chinese nationals working overseas in VBD endemic countries. Screening of locals for VBDs such as Malaria also takes place for individuals working in and around a Chinese factory. As an example, yellow fever vaccination and VBD screening programs take place on the African continent where populations of Chinese economic migrants work within the extractive industries.

*“An overseas factory must carry out such kind of work locally and when they need expert support to deal with any medical situation, we can go with them to deal with it locally.” [R3]*

Beijing hospital workforce staff are contracted and deployed to overseas countries to deliver vaccines to Chinese nationals. Regarding IHR (2005), it was found that national level public health authorities were more familiar with the regulations than district and provincial authorities. Domestic guidelines are compatible with WHO regulations and modified with China specific content to suit the Chinese context. Position papers are published to inform about adaptations. At the national level, China CDC releases monthly reports, which include WHO content and modified suggestions [R6]. Provincial and district level CDCs follow these adapted guidelines to suit the Chinese setting.

## 4. Discussion

This study elucidated experiences and perceptions of local stakeholders regarding Beijing’s VBD surveillance system. A summary of our main findings is presented in Box 1.

Box 1. **Main findings of the results.**

**Main findings**

- Beijing’s VBD surveillance operates through a bottom to top reporting structure in line with national guidelines. Human case reporting (NIDRIS) and vector surveillance (NVSN) function within separate reporting networks;
- Stakeholders deemed Beijing’s VBD surveillance to be operationally straightforward, specific, representative, timely, accepted by experts, flexible and adaptable;
- Challenges in Beijing’s VBD surveillance included doubtful sensitivity at population level (dependent on citizens to seek health care), questionable reliability (lack of reporting requirements for private hospitals) and a lack of VBD outbreaks to test system efficacy;
- Response guidelines for dengue and Japanese encephalitis outbreaks are found at national level, but no predefined outbreak plans exist for non-endemic VBDs
- A gap in Lyme disease identification was indicated;
- Planned management procedures were in place for vector control measures;
- Feedback mechanisms facilitate early warning of and timely response to outbreaks.

Beijing’s health system functions to a top-down governance framework, with syndromic and vector surveillance data shared in bottom-up communication lines for analysis. This top-down approach and system layout render Beijing VBD SRS both rigid and dynamic. The current system promotes routine surveillance as well as adaptability and flexibility towards sudden changes due to syndromic surveillance features. Surveillance systems that are simple, flexible, acceptable, and stable, will increase comprehensiveness and utility of public health actions [27]. When considered in the context of complex environmental and human pressures that influence the distribution of vectors and pathogen transmission, Beijing’s VBD SRS can support a timely response.

An important feature of Beijing’s VBD SRS is syndromic surveillance. Patterson and Durrheim (2003) found that syndromic surveillance is able to adapt to rapidly shifting public health needs and has the flexibility to utilise different approaches depending on the situation [28]. This adaptability makes syndromic surveillance a highly relevant public health tool. Additionally, entomological components necessary for optimal VBD SRS are integrated into Beijing’s VBD SRS and follow an almost identical reporting structure as that of human disease cases. Braks et al. (2011) [2] argues that improving the mutual understanding and communications between medical entomology and public health supports the development of desirable interventions to VBD outbreaks [2]. Nevertheless, challenges remain. Beijing’s passive syndromic surveillance relies on an assumed self-agency of patients to enter the health system. Without the intent to seek healthcare, a patient presenting with VBD syndromes would not be identified by the system. Thus, sensitivity at the population level is sub-optimal compared to when a patient is already at the health centre, as reported by Beijing’s experts. However, should an outbreak occur, or the risk of vector spread and disease transmission increase, an active surveillance strategy pivot could be enabled by public health authorities in Beijing to purposefully identify cases [29].

VBD response in Beijing is believed to be sufficient, with Beijing’s response to Zika cases in 2016 used as an example by Beijing’s experts. National management plans were available to support timely and harmonised interventions. However, as this study revealed, occasions for health system outbreak response toward VBDs are not common. In China’s tropical south, municipalities observe a greater and more complex VBD reality, specifically their distribution, prioritisation, and SRS. This difference is due to climatic and geographic variables that are not present in Beijing [30,31]. As opportunities for health system response are rare, research and evaluation of control actions contribute in a minimal manner to control actions for VBD interventions in Beijing. Despite an apparent lack of opportunity to practice and train response systems, the health system seems prepared to deliver rapid and strict interventions when necessary. National governance structures and government leadership underpin this, supporting adaptability of the VBD outbreak response pathway from the top down. This enables flexibility to deal with acute VBD public health threats.

Although climate and environmental factors are known to influence the distribution of vectors and the pathogens they transmit, infrequent collaborations were made between public health and meteorological institutes. When collaborations did occur, action was isolated to research projects dependent on inter-sectorial agreement. Yet, routine environment and climate monitoring for VBD SRS have not been optimised in Beijing. Considering such signals contribute to the transmission and distribution patterns of VBDs, inclusion of such signals could enhance VBD SRS [2]. Putting such research into routine practice could benefit VBD SRS where indicators beyond the prioritised disease and vector signals may be operationalised. This would embrace a One Health approach, as shown in the work of Ceccato et al. (2018) through their WHO/TDR-IDRC Research Initiative on VBDs and Climate Change project [32].

It has been observed that a centralised health system permits opportunities to enforce policy with little compromise and delay, supporting system flexibility and adaptability. Smullen and Kai Hong (2015), in their paper that compares health systems in Asian countries, found that centralised and unified health systems are able to deliver system functions and pursue improvements free from opposition by political and administrative levels, or by the private sector [33]. Beijing’s health system provides effective regulation backed up by legal and stern enforcement measures to maintain stability. This construct present in Beijing allows flexibility, once central authorities have agreed to suggestions and novel ideas.

Although Internet based surveillance networks are integrated into VBD SRS, technological advancements have also enabled the use of spatial-temporal modelling for VBD prevention and control. Ceccato et al. (2018) state that important progress has been made in the monitoring of the weather, climate, environmental and anthropogenic factors that influence the reduction or the re-emergence of VBDs. GIS and remote sensing are also improving knowledge of climatic, environmental, and biodiversity factors influencing vector-borne diseases [32]. Surveillance data can be augmented by combining interacting scales of effect for biological system (host, vector, human disease case) that contribute to VBD dynamics [34]. These operate to their own explicit temporal and spatial scale. Modelling using GIS captures these dynamics to provide unique insight into the spatial and temporal attributes of VBDs. However, the comprehensive use of GIS applications for VBD in Beijing was not identified. This study revealed that infrastructure was not a limiting factor, but that restricted availability of qualified technicians prevented integration. Considering opportunities exist in the understanding of endemic VBDs, increasing the capacity of spatial-temporal analysis might prove beneficial to understand VBD dynamics, supporting effective prevention and control.

Although the system in Beijing is seen as relevant, functional, and in agreement with the IHR for the context it exists in, the interviews also provided insights regarding Lyme disease that may be relevant for the wider context of arbovirus surveillance. Dou et al. (2015) identified a 5.1% seroprevalence in Beijing’s rural population, indicating endemic status [35]. The current study finds that the health system rarely identifies positive cases through routine monitoring, with those affected scarcely diagnosed or cured. The passive syndromic surveillance relies on individual agency to enter the health system. As ticks are not observed in Beijing’s urban core, there is no perceived need to look for them, resulting in a low sensitivity of the surveillance system to this vector. There is limited action in endemic rural Beijing as no obligatory notification system is available for Lyme disease. Laboratory testing is restricted for Lyme disease beyond reference laboratories, and late presentation to the health system is a limiting factor for treatment contributing to under diagnosis and progressive chronic disease burdens [35]. In line with Dou et al. (2015), public health in Beijing may find solace in promoting basic pre-emptive knowledge in rural settings about ticks and Lyme disease to the public, including occupational risks, supporting health system actions toward VBD SRS. Further, the challenge of underreporting of human cases forms an argument for enhanced commitment to arboviral surveillance in animals. Arboviruses affecting human health are zoonotic and originate from transmission cycles involving wild animals as reservoir and possible amplification hosts. Spill over transmission from animal reservoirs to human reservoirs can have widespread public health implications, both within the initial urban area as through global travel routes, and epidemic outbreaks may result in a permanent endemic status [36]. As monitoring the presence of arboviruses potentially harmful for human health in animals may aid early warning of VBD occurrence, an integrated VBD surveillance including both human and animal cases is of particular importance in rural areas surrounding large metropoles.

VBDs discussed in this study do not pose an immediate threat to the province of Beijing compared to other infectious diseases. To provide qualitative research representing government stakeholders was the aim of our study and is therefore as well seen as the strength to shed further light on understanding the surveillance and response system on VBD in the Beijing province. We focused on experts from prominent public health institutions in Beijing, mainly CDC offices in the urban core. Despite our inclusion of offices from rural areas, future assessments in Beijing will need to strategically include a greater number of experts from rural settings. A limitation of our study can be seen with not representing non-governmental stakeholders. Further work in this area could focus on including private and non-government organisations and groups to enhance perspectives and understanding of VBD SRS in Beijing. Additionally, our study was conducted over a short period of time that may have prevented experts interested in participating to do so. Our sample size of only 11 expert interviews can lead to bias and further research in this setting with more time for data collection to increase the diversity of respondents and views would be recommended.

## 5. Conclusions

Findings reveal that core SRS capacities are compatible to the IHR, with constructive opportunities apparent for further development. Recommendations include: (1) Refine current surveillance and monitoring systems for VBDs of unknown distribution. (2) Increase public educational strategies utilising system strengths in policies setting and enforcement, including those of Chinese nationals working overseas in VBD endemic countries. (3) Provide specific actions toward multidisciplinary approaches to VBD SRS, developing inter-sectorial approaches and coordination to VBD risk for routine surveillance. (4) Increase awareness and mobilisation of civil society toward vector populations. What emerges from the study is a central governance framework involving both technical and administrative health authorities at various levels that provide direction and coordination for VBD SRS. This top-down governance also entails opportunities for the integration of other institutions thus posing system flexibility. The integration of dedicated web-portals, automated alert systems and syndromic surveillance contributes to effective dissemination of data to enable timely response, although rare, when needed.

## Figures and Tables

**Table 1:** Characteristics of experts who participated in this study. (*) Indicates use of a translator during one-to-one interviews.

Respondent	Governance Level	Institution	Profession	Interview Focus
R 1	Province	Infectious Disease & Endemic Disease	Epidemiologist	Vector Borne Diseases
R 2	Province	Infectious Disease & Endemic Disease	Parasitologist	Malaria (*)
R 3	Province	Hospital	Clinician	Primary Care
R 4	District	Chaoyang CDC	Epidemiologist	Vector Borne Diseases
R 5	Province	Infectious Disease & Endemic Disease	Epidemiologist	Lyme
R 6	District	Shun Yi CDC	Professor	Vector Borne Diseases
R 7	Province	Immunisation & Prevention	Immunologist	Japanese encephalitis (*)
R 8	National	Communicable Disease Control & Prevention	Assistant Professor	Vector Borne Diseases
R 9	Province	Disinfection & Vector Control	Entomologist	Vectors
R 10	Province	Infectious Disease & Endemic Disease	Epidemiologist	Surveillance Systems
R 11	Province	Infectious Disease & Endemic Disease	Laboratory Scientist	Vector Borne Diseases

## Supplementary Materials

The following are available online at https://www.mdpi.com/1660-4601/17/22/8512/s1. Supplementary Material 1: Interview guide (guiding questions).

## Abbreviations

CCHF	Crimean-Congo haemorrhagic fever
CDC	Center for Disease Prevention and Control
CIDARS	China Infectious Disease Automated-alert and Response System
IHR	International Health Regulation
MoU	Memorandum of Understanding
NIDRIS	National Infectious Disease Reporting Information System in China
NVSN	National Vector Surveillance Networks
SARS	Sever Acute Respiratory Syndrome
SMS	Short Message Service
SRS	Surveillance and Response System
VBD	Vector-Borne Disease
