# Supplementary material for: Exploring Vector-Borne Disease Surveillance and Response Systems in Beijing, China: A Qualitative Study from the Health System Perspective
Source: Int J Environ Res Public Health. 2020 Nov 17;17(22):8512. doi: 10.3390/ijerph17228512 (PMC7698447; doi:10.3390/ijerph17228512)
Supplement: Supplementary file 1 [file ijerph-17-08512-s001.pdf]

Supplementary material 1: Interview guide (guiding questions)

The aim of this research is to get an overview of the surveillance and response of vectors and vector-borne diseases (VBDs) in the municipality of Beijing, and to describe points of strength and of improvement, and local health system practices.

VBD Monitoring

1. How does monitoring work?
    a. Is monitoring being done? How is it being performed?
    b. Are all five indicators (vector, pathogen, disease, environment and climate) being monitored in Beijing? How?
2. Looking at the following hierarchy chart (figure 1), Which type of surveillance is currently present for VBD?
    a. Can you explain how these types of surveillance work in Beijing?
    b. Who monitors, where is data being gathered and who processes the data?

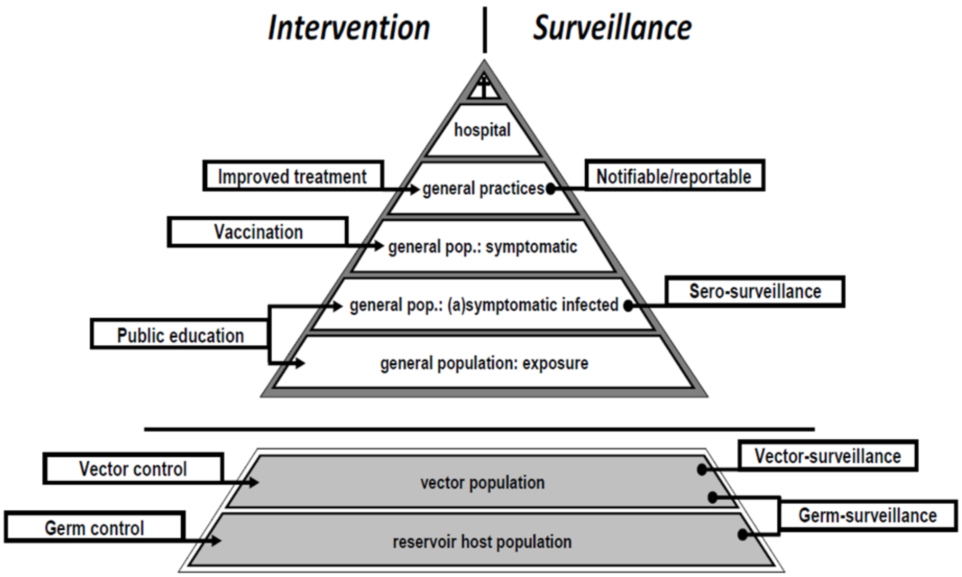

Figure 1: Source: (Braks et al., 2011)

3. Looking at the following Performance of Surveillance Systems box (figure 2), can you answer these nine questions for the surveillance of the five monitoring indicators (vector, pathogen, disease, environment and climate)?

Performance of the Surveillance System

1. In your opinion, how complex is the surveillance system (in structure and ease of operation)? - *(simplicity)*
2. In your opinion, how sensitive is the surveillance system (what is the chance that an exotic mosquito is found when there is a low density)? - *(sensitivity)*
3. Is the surveillance system specific? – *(specificity/ predictive value positive)*
4. In your opinion, how complete and valid is the data used in the surveillance system? - *(quality)*
5. In your opinion, how well does the data represent the real situation (type, place, time)? - *(representativeness)*
6. In your opinion, how reliable (consistent) is the method of surveillance data collection? - *(stability)*
7. In your opinion, how quick does the surveillance system operate? (is data available soon enough to undertake effective action?) - *(timeliness)*
8. Are organization willing to participate in the surveillance system? - *(acceptability)*
9. In your opinion, how flexible is the surveillance system to adapt to changing conditions ? - *(flexibility)*

Figure 2: Source: (CDC, 2001)

Research

4. Is scientific research being conducted?
    a. What type of research is conducted?
    b. What knowledge does the research produce?
    c. Who conducts this research?

Disease burden

5. Are agreements made for harmonization?
6. Are agreements made for prioritizing VBDs in Beijing?

Public Health Authority

7. Which public health authority receives the monitoring data and how is it sent?
    a. What is being done with the data?
    b. How do these authorities communicate?
    c. Are all organisations that should be involved with surveillance and response of VBD involved?
8. How would you describe legislation, guidelines or outbreak management plans that are relevant for the surveillance of VBD?

Decision to Take Action

9. How are decisions being made?
    a. Who is responsible for making decisions?
    b. How would you describe the types of interventions being implemented?
10. Can you describe the response for each level of intervention, looking at the pyramid (figure 2)? In your opinion, is the response in Beijing sufficient?
11. How would you describe the overall response system for VBD?

R&D Control Actions

12. Is research being conducted to investigate the effects of implemented interventions (for the five indicators)?
    a. Is the feedback of this research being used?
    b. Who conducts this research?
